# Bis(1-carbamimidoyl-2-ethyl­isourea)copper(II) bis­(perchlorate)

**DOI:** 10.1107/S1600536809034965

**Published:** 2009-09-05

**Authors:** Atittaya Meenongwa, Unchulee Chaveerach, Claire Wilson, Alexander J. Blake

**Affiliations:** a Department of Chemistry and Center of Excellence for Innovation in Chemistry, Faculty of Science, Khon Kaen University, Khon Kaen 40002, Thailand; b School of Chemistry, University of Nottingham, University Park, Nottingham NG7 2RD, England

## Abstract

The title complex, [Cu(C_4_H_10_N_4_O)_2_](ClO_4_)_2_ or [Cu(*L* ^1*e*^)_2_](ClO_4_)_2_, where *L* ^1*e*^ is 1-carbamimidoyl-2-ethyl­isourea, was obtained from the ethano­lysis reaction of 2-cyano­guanidine and copper(II) perchlorate hexa­hydrate in a 2:1 molar ratio. The two bidentate *L* ^1*e*^ ligands are coordinated to the Cu^II^ center through N-donor atoms, leading to the CuN_4_ chromophore. The Cu^II^ environment is slightly distorted square-planar, with a dihedral angle of 5.17 (6)° between the two six-membered chelate rings. One of the ClO_4_^−^ anions is disordered over two positions in a 0.6:0.4 ratio.

## Related literature

For general reviews of 2-alkyl-1-carbamimidoyl­isourea ligand systems, see Hubberstey *et al.* (2000 ▶); Singh *et al.* (2005 ▶). For a previous study of copper(II) complexes containing the 1-carbamimidoyl-2-ethyl­isourea ligand in a 1:1 molar ratio, see Begley *et al.* (1986 ▶).
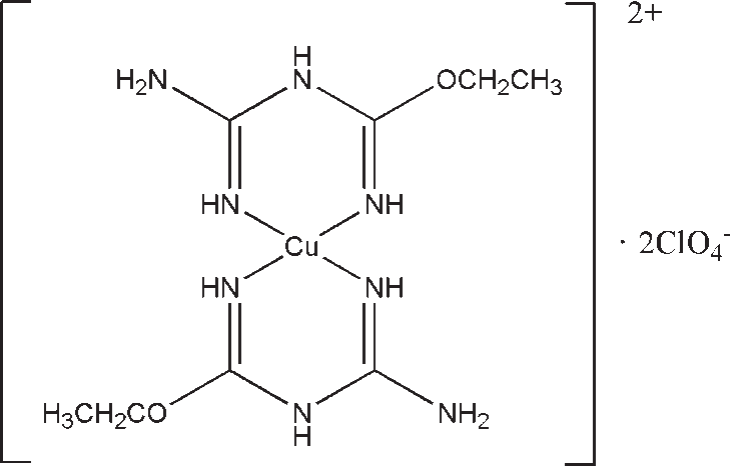


## Experimental

### 

#### Crystal data

[Cu(C_4_H_10_N_4_O)_2_](ClO_4_)_2_
*M* *_r_* = 522.76
Monoclinic, 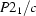

*a* = 10.6928 (7) Å
*b* = 13.8061 (9) Å
*c* = 13.5977 (9) Å
β = 102.657 (1)°
*V* = 1958.6 (2) Å^3^
*Z* = 4
Mo *K*α radiation
μ = 1.46 mm^−1^
*T* = 150 K
0.42 × 0.15 × 0.10 mm

#### Data collection

Bruker SMART APEX CCD area-detector diffractometer
Absorption correction: multi-scan (*SADABS*; Sheldrick, 1996 ▶) *T*_min_ = 0.846, *T*_max_ = 1.000
16840 measured reflections
4516 independent reflections
3738 reflections with *I* > 2σ(*I*)
*R*_int_ = 0.015

#### Refinement

*R*[*F* ^2^ > 2σ(*F* ^2^)] = 0.034
*wR*(*F* ^2^) = 0.110
*S* = 1.02
4516 reflections
298 parameters
45 restraints
H-atom parameters constrained
Δρ_max_ = 0.77 e Å^−3^
Δρ_min_ = −0.30 e Å^−3^

### 

Data collection: *SMART* (Bruker, 2001 ▶); cell refinement: *SAINT* (Bruker, 2000 ▶); data reduction: *SAINT* and *SHELXTL* (Sheldrick, 2008 ▶); program(s) used to solve structure: *SHELXS97* (Sheldrick, 2008 ▶); program(s) used to refine structure: *SHELXL97* (Sheldrick, 2008 ▶); molecular graphics: *DIAMOND* (Brandenburg, 2005 ▶); software used to prepare material for publication: *enCIFer* (Allen *et al.*, 2004 ▶) and *publCIF* (Westrip, 2009 ▶).

## Supplementary Material

Crystal structure: contains datablocks global, I. DOI: 10.1107/S1600536809034965/jh2102sup1.cif

Structure factors: contains datablocks I. DOI: 10.1107/S1600536809034965/jh2102Isup2.hkl

Additional supplementary materials:  crystallographic information; 3D view; checkCIF report

## Figures and Tables

**Table:** 

Cu1—N1	1.9411 (15)
Cu1—N4	1.9606 (15)
Cu1—N5	1.9376 (15)
Cu1—N8	1.9641 (15)

**Table:** 

N1—Cu1—N4	89.00 (6)
N1—Cu1—N5	177.92 (5)
N1—Cu1—N8	90.83 (7)
N4—Cu1—N5	90.95 (7)
N4—Cu1—N8	178.47 (6)
N5—Cu1—N8	89.27 (7)

**Fig. 1.**
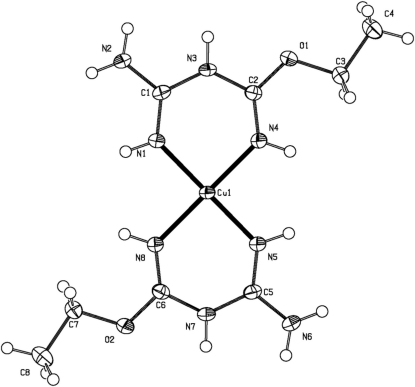
View of the title complex, showing the atom numbering of the cationic [Cu(L1e)2]2+ moiety of [Cu(L1e)2](ClO4)2. Displacement ellipsoids are drawn at the 50% probability level.

**Fig. 2.**
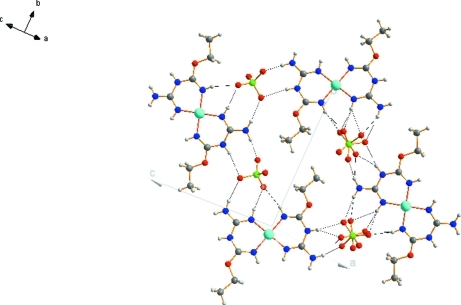
A view of the crystal packing along the b axis. Hydrogen bonds are shown as a dashed lines.

## supplementary crystallographic information

### Comment

Copper(II) complexes contaning ligands based on 2-alkyl-1-carbamimidoylisourea have been considered to be of importance in crystal engineering (Hubberstey *et al.*, 2000). This ligand system shows versatile hydrogen bonding capability which is deployed to construct extended architectures. Moreover, it also exhibits growth inhibitory activity against pathogenic fungi and bacteria (Singh *et al.*, 2005). Ethanolysis of the cyanoguanidine precursor in the presence of CuCl_2_ in a 1:1 molar ratio has been previously reported by Begley *et al.* (1986). The crystal structure of this product reveals that the mono-chelate complex is comprised solely of dinuclear [Cu(*L*^1^*^e^*)Cl_2_]_2_ units (*L*^1^*^e^* = 1-carbamimidoyl-2-ethylisourea) in which each Cu^II^ center is bridged by the neighboring chloride in the axial position to give a square-pyramidal geometry.

Herein, we present the structure of a copper(II) complex containing the same bidentate *L*^1^*^e^* ligand but with the charge balance provided by two perchlorate anions instead of the chloride anions as described above. The [Cu(*L*^1^*^e^*)_2_](ClO_4_)_2_ was obtained from the similar procedure as previously reported by Begley *et al.* (1986), but using a 2:1 molar ratio of 2-cyanoguanidine to copper(II) perchlorate hexahydrate. Structure determination on the title product reveals a [Cu(*L*^1^*^e^*)_2_]^2+^ cation and two ClO_4_^-^ counter anions. Fig. 1 shows the [Cu(*L*^1^*^e^*)_2_]^2+^ unit. The Cu^II^ center is coordinated by two *N*,*N*-bidentate ligands, thus yielding a slightly distorted square-planar CuN_4_ geometry (Table 1) with Cu—N bond distances of 1.9376 (15)–1.9641 (15) Å. The bite angles of 89.00 (6)° for N1—Cu1—N4 and 89.27 (7) ° for N1—Cu1—N8 are slightly less than 90° with a dihedral angle of 5.17 (6) ° between the two six-membered chelate rings. Additionally, the intermolecular hydrogen bonds of the type N—H···O (perchlorate) also stabilize the [Cu(*L*^1^*^e^*)_2_]^2+^ cation to give a two-dimentional layered structure (Fig.2)

### Experimental

Suitable single crystals of the title complex were raised from the initial product, which was obtained from the ethanolysis reaction of 2-cyanoguanidine precursor (0.1682 g, 2 mmol, Aldrich, 99%) in the presence of copper(II) perchlorate hexahydrate (0.3705 g, 1 mmol,Aldrich, 98%). The reaction was carried out in the refluxing condition for 24 h, then cooled down and filtered off to remove excess solid. The reddish-pink powder was obtained by reducing the solvent volume. The pink blocky single crystals were grown by slow vapor phase diffusion of diethylether into the methanolic solution of this product at room temperature over a few days.

### Refinement

H atoms were positioned geometrically and refined as riding atoms, with N—H = 0.88, *C*—*H*(methyl) = 0.98 and *C*—*H*(methylene) = 0.99 Å, and with *U*_iso_(H) = *xU*_eq_(C, N), where *x* = 1.5 for methyl H atoms and 1.2 for all others.

### Crystal data

**Table:**

[Cu(C_4_H_10_N_4_O)_2_](ClO_4_)_2_	*F*(000) = 1068
*M**_r_* = 522.76	*D*_x_ = 1.773 Mg m^−^^3^
Monoclinic, *P*2_1_/*c*	Mo *K*α radiation, λ = 0.71073 Å
Hall symbol: -P 2ybc	Cell parameters from 6505 reflections
*a* = 10.6928 (7) Å	θ = 2.8–27.6°
*b* = 13.8061 (9) Å	µ = 1.46 mm^−^^1^
*c* = 13.5977 (9) Å	*T* = 150 K
β = 102.657 (1)°	Block, pink
*V* = 1958.6 (2) Å^3^	0.42 × 0.15 × 0.10 mm
*Z* = 4	

### Data collection

**Table:**

Bruker SMART APEX CCD area-detector diffractometer	4516 independent reflections
Radiation source: sealed tube	3738 reflections with *I* > 2σ(*I*)
graphite	*R*_int_ = 0.015
ω scans	θ_max_ = 27.6°, θ_min_ = 2.5°
Absorption correction: multi-scan (*SADABS*; Sheldrick, 1996)	*h* = −13→13
*T*_min_ = 0.846, *T*_max_ = 1.000	*k* = −17→17
16840 measured reflections	*l* = −17→17

### Refinement

**Table:**

Refinement on *F*^2^	Primary atom site location: structure-invariant direct methods
Least-squares matrix: full	Secondary atom site location: difference Fourier map
*R*[*F*^2^ > 2σ(*F*^2^)] = 0.034	Hydrogen site location: inferred from neighbouring sites
*wR*(*F*^2^) = 0.110	H-atom parameters constrained
*S* = 1.02	*w* = 1/[σ^2^(*F*_o_^2^) + (0.073*P*)^2^ + 0.715*P*] where *P* = (*F*_o_^2^ + 2*F*_c_^2^)/3
4516 reflections	(Δ/σ)_max_ = 0.003
298 parameters	Δρ_max_ = 0.77 e Å^−^^3^
45 restraints	Δρ_min_ = −0.30 e Å^−^^3^

### Special details

**Table:**

Geometry. All e.s.d.'s (except the e.s.d. in the dihedral angle between two l.s. planes) are estimated using the full covariance matrix. The cell e.s.d.'s are taken into account individually in the estimation of e.s.d.'s in distances, angles and torsion angles; correlations between e.s.d.'s in cell parameters are only used when they are defined by crystal symmetry. An approximate (isotropic) treatment of cell e.s.d.'s is used for estimating e.s.d.'s involving l.s. planes.
Refinement. Refinement of *F*^2^ against ALL reflections. The weighted *R*-factor *wR* and goodness of fit *S* are based on *F*^2^, conventional *R*-factors *R* are based on *F*, with *F* set to zero for negative *F*^2^. The threshold expression of *F*^2^ > σ(*F*^2^) is used only for calculating *R*-factors(gt) *etc*. and is not relevant to the choice of reflections for refinement. *R*-factors based on *F*^2^ are statistically about twice as large as those based on *F*, and *R*- factors based on ALL data will be even larger.

### Fractional atomic coordinates and isotropic or equivalent isotropic displacement parameters (Å^2^)

**Table:**

	*x*	*y*	*z*	*U*_iso_*/*U*_eq_	Occ. (<1)
Cu1	0.255629 (17)	0.501463 (12)	0.002819 (13)	0.01863 (11)	
O1	0.34227 (13)	0.23854 (9)	−0.12296 (10)	0.0234 (3)	
O2	0.15463 (13)	0.76578 (10)	0.11725 (11)	0.0263 (3)	
N1	0.37087 (15)	0.53760 (11)	−0.08322 (11)	0.0220 (3)	
H1A	0.3881	0.5999	−0.0842	0.026*	
N2	0.51010 (18)	0.51972 (13)	−0.19225 (14)	0.0287 (4)	
H2A	0.5290	0.5818	−0.1892	0.034*	
H2B	0.5455	0.4807	−0.2295	0.034*	
N3	0.40771 (15)	0.38571 (12)	−0.14711 (12)	0.0236 (3)	
H3C	0.4459	0.3549	−0.1890	0.028*	
N4	0.27510 (15)	0.36547 (11)	−0.03258 (12)	0.0240 (3)	
H4D	0.2374	0.3228	−0.0007	0.029*	
N5	0.13542 (15)	0.46577 (12)	0.08450 (12)	0.0228 (3)	
H5A	0.1221	0.4032	0.0887	0.027*	
N6	−0.01529 (18)	0.48529 (13)	0.18399 (15)	0.0308 (4)	
H6A	−0.0308	0.4227	0.1839	0.037*	
H6B	−0.0562	0.5251	0.2165	0.037*	
N7	0.08417 (15)	0.61897 (11)	0.13659 (12)	0.0239 (3)	
H7C	0.0341	0.6511	0.1685	0.029*	
N8	0.24037 (15)	0.63742 (11)	0.04106 (12)	0.0234 (3)	
H8D	0.2902	0.6788	0.0187	0.028*	
C1	0.42637 (17)	0.48409 (14)	−0.13953 (14)	0.0212 (4)	
C2	0.33594 (16)	0.33037 (13)	−0.09646 (13)	0.0196 (3)	
C3	0.2658 (2)	0.16826 (14)	−0.08128 (15)	0.0271 (4)	
H3A	0.1736	0.1848	−0.1010	0.033*	
H3B	0.2918	0.1673	−0.0068	0.033*	
C4	0.2908 (3)	0.07137 (15)	−0.12418 (18)	0.0394 (5)	
H4A	0.2415	0.0213	−0.0983	0.059*	
H4B	0.3824	0.0562	−0.1042	0.059*	
H4C	0.2647	0.0736	−0.1979	0.059*	
C5	0.07131 (18)	0.52029 (14)	0.13420 (14)	0.0213 (4)	
C6	0.16667 (17)	0.67339 (13)	0.09459 (13)	0.0208 (4)	
C7	0.23538 (19)	0.83607 (13)	0.08012 (15)	0.0263 (4)	
H7A	0.2148	0.8375	0.0055	0.032*	
H7B	0.3271	0.8195	0.1039	0.032*	
C8	0.2064 (2)	0.93278 (15)	0.12204 (18)	0.0367 (5)	
H8A	0.2581	0.9832	0.0994	0.055*	
H8B	0.2271	0.9300	0.1959	0.055*	
H8C	0.1152	0.9478	0.0981	0.055*	
Cl1	0.57714 (4)	0.30297 (3)	−0.35071 (3)	0.02420 (13)	
O1C	0.56080 (16)	0.24917 (11)	−0.44314 (11)	0.0352 (3)	
O2C	0.70647 (16)	0.33555 (14)	−0.32002 (14)	0.0485 (4)	
O3C	0.49245 (17)	0.38564 (12)	−0.36660 (15)	0.0479 (4)	
O4C	0.5435 (2)	0.24293 (13)	−0.27344 (13)	0.0484 (4)	
Cl2	0.07496 (4)	0.20306 (3)	0.14733 (3)	0.02688 (13)	
O5C	−0.0235 (2)	0.27739 (17)	0.1328 (2)	0.0474 (7)	0.60
O6C	0.1007 (2)	0.1677 (2)	0.24675 (19)	0.0505 (8)	0.60
O7C	0.1915 (3)	0.2497 (2)	0.1312 (2)	0.0464 (7)	0.60
O8C	0.0365 (4)	0.1307 (2)	0.0748 (3)	0.0683 (11)	0.60
O5B	0.0565 (5)	0.2553 (3)	0.0573 (3)	0.0443 (10)	0.40
O6B	0.0400 (5)	0.2556 (3)	0.2287 (3)	0.0477 (11)	0.40
O7B	−0.0058 (4)	0.1143 (3)	0.1295 (4)	0.0393 (10)	0.40
O8B	0.2052 (5)	0.1690 (4)	0.1763 (4)	0.0613 (14)	0.40

### Atomic displacement parameters (Å^2^)

**Table:**

	*U*^11^	*U*^22^	*U*^33^	*U*^12^	*U*^13^	*U*^23^
Cu1	0.02119 (16)	0.01737 (16)	0.01994 (16)	0.00250 (7)	0.01016 (11)	−0.00086 (8)
O1	0.0291 (7)	0.0187 (6)	0.0247 (6)	0.0015 (5)	0.0109 (5)	−0.0030 (5)
O2	0.0329 (7)	0.0189 (6)	0.0309 (7)	0.0044 (5)	0.0153 (6)	−0.0005 (5)
N1	0.0262 (8)	0.0184 (8)	0.0245 (8)	0.0013 (6)	0.0120 (6)	−0.0023 (6)
N2	0.0347 (9)	0.0245 (8)	0.0340 (10)	−0.0030 (7)	0.0232 (8)	−0.0044 (7)
N3	0.0273 (8)	0.0225 (8)	0.0258 (8)	−0.0006 (6)	0.0159 (6)	−0.0060 (6)
N4	0.0312 (8)	0.0185 (7)	0.0264 (8)	0.0023 (6)	0.0153 (6)	0.0011 (6)
N5	0.0273 (8)	0.0193 (8)	0.0255 (8)	0.0015 (6)	0.0137 (6)	−0.0012 (6)
N6	0.0348 (9)	0.0272 (8)	0.0382 (10)	−0.0007 (7)	0.0249 (8)	−0.0021 (7)
N7	0.0255 (8)	0.0223 (8)	0.0283 (8)	0.0035 (6)	0.0152 (6)	−0.0028 (6)
N8	0.0268 (8)	0.0188 (7)	0.0284 (8)	0.0011 (6)	0.0145 (6)	−0.0002 (6)
C1	0.0190 (8)	0.0256 (9)	0.0209 (9)	0.0012 (7)	0.0086 (7)	−0.0011 (7)
C2	0.0210 (8)	0.0192 (8)	0.0192 (8)	0.0021 (6)	0.0054 (6)	−0.0016 (6)
C3	0.0359 (10)	0.0200 (9)	0.0282 (10)	−0.0006 (7)	0.0131 (8)	−0.0006 (7)
C4	0.0589 (15)	0.0227 (10)	0.0404 (12)	−0.0013 (9)	0.0192 (11)	−0.0065 (9)
C5	0.0207 (8)	0.0245 (8)	0.0207 (9)	0.0023 (7)	0.0087 (7)	−0.0007 (7)
C6	0.0230 (8)	0.0190 (8)	0.0204 (8)	0.0034 (6)	0.0050 (6)	−0.0014 (7)
C7	0.0323 (10)	0.0200 (9)	0.0283 (10)	0.0010 (7)	0.0100 (8)	0.0001 (7)
C8	0.0469 (13)	0.0233 (10)	0.0413 (12)	0.0019 (9)	0.0127 (10)	−0.0068 (9)
Cl1	0.0286 (2)	0.0230 (2)	0.0226 (2)	−0.00094 (16)	0.00904 (17)	−0.00272 (16)
O1C	0.0452 (9)	0.0319 (8)	0.0297 (8)	0.0036 (6)	0.0106 (6)	−0.0061 (6)
O2C	0.0362 (9)	0.0562 (11)	0.0490 (10)	−0.0109 (8)	0.0002 (7)	0.0025 (9)
O3C	0.0492 (10)	0.0360 (9)	0.0576 (11)	0.0121 (7)	0.0098 (8)	−0.0116 (8)
O4C	0.0669 (12)	0.0479 (10)	0.0355 (9)	−0.0089 (9)	0.0222 (8)	0.0061 (8)
Cl2	0.0333 (3)	0.0238 (2)	0.0238 (2)	0.00216 (17)	0.00677 (18)	0.00258 (17)
O5C	0.0423 (15)	0.0277 (13)	0.0681 (19)	0.0100 (11)	0.0033 (13)	−0.0050 (12)
O6C	0.0375 (14)	0.078 (2)	0.0338 (14)	−0.0190 (14)	0.0026 (11)	0.0286 (14)
O7C	0.0493 (16)	0.0528 (17)	0.0457 (16)	0.0050 (13)	0.0293 (13)	0.0186 (13)
O8C	0.081 (3)	0.0444 (18)	0.063 (2)	0.0169 (17)	−0.0195 (19)	−0.0222 (17)
O5B	0.062 (3)	0.034 (2)	0.037 (2)	−0.0087 (19)	0.0114 (19)	0.0070 (17)
O6B	0.066 (3)	0.042 (2)	0.047 (3)	−0.010 (2)	0.039 (2)	−0.015 (2)
O7B	0.052 (3)	0.026 (2)	0.041 (2)	−0.0100 (17)	0.014 (2)	0.0061 (17)
O8B	0.040 (2)	0.072 (3)	0.065 (3)	0.014 (2)	−0.004 (2)	−0.022 (3)

### Geometric parameters (Å, °)

**Table:**

Cu1—N1	1.9411 (15)	Cl2—O6C	1.406 (2)
Cu1—N4	1.9606 (15)	Cl2—O6B	1.439 (4)
Cu1—N5	1.9376 (15)	Cl2—O8B	1.441 (5)
Cu1—N8	1.9641 (15)	Cl2—O5C	1.453 (2)
O1—C2	1.324 (2)	Cl2—O7C	1.461 (3)
O1—C3	1.461 (2)	Cl2—O7B	1.488 (4)
O2—C6	1.325 (2)	N1—H1A	0.8800
O2—C7	1.461 (2)	N2—H2A	0.8800
N1—C1	1.297 (2)	N2—H2B	0.8800
N2—C1	1.356 (2)	N3—H3C	0.8800
N3—C2	1.371 (2)	N4—H4D	0.8800
N3—C1	1.373 (2)	N5—H5A	0.8800
N4—C2	1.288 (2)	N6—H6A	0.8800
N5—C5	1.302 (2)	N6—H6B	0.8800
N6—C5	1.351 (3)	N7—H7C	0.8800
N7—C5	1.369 (2)	N8—H8D	0.8800
N7—C6	1.375 (2)	C3—H3A	0.9900
N8—C6	1.284 (2)	C3—H3B	0.9898
C3—C4	1.506 (3)	C4—H4A	0.9802
C7—C8	1.510 (3)	C4—H4B	0.9800
Cl1—O2C	1.4269 (17)	C4—H4C	0.9804
Cl1—O1C	1.4372 (15)	C7—H7A	0.9902
Cl1—O3C	1.4435 (16)	C7—H7B	0.9898
Cl1—O4C	1.4445 (16)	C8—H8A	0.9796
Cl2—O5B	1.397 (4)	C8—H8B	0.9807
Cl2—O8C	1.400 (3)	C8—H8C	0.9802
			
N1—Cu1—N4	89.00 (6)	C6—N7—H7C	116.4
N1—Cu1—N5	177.92 (5)	C6—N8—H8D	115.9
N1—Cu1—N8	90.83 (7)	Cu1—N8—H8D	115.9
N4—Cu1—N5	90.95 (7)	O1—C3—H3A	110.5
N4—Cu1—N8	178.47 (6)	C4—C3—H3A	110.4
N5—Cu1—N8	89.27 (7)	O1—C3—H3B	110.5
C2—O1—C3	117.66 (14)	C4—C3—H3B	110.5
C6—O2—C7	117.66 (15)	H3A—C3—H3B	108.7
C1—N1—Cu1	129.95 (14)	C3—C4—H4A	109.5
C2—N3—C1	126.99 (15)	C3—C4—H4B	109.4
C2—N4—Cu1	128.47 (13)	H4A—C4—H4B	109.5
C5—N5—Cu1	129.88 (14)	C3—C4—H4C	109.5
C5—N7—C6	127.18 (16)	H4A—C4—H4C	109.5
C6—N8—Cu1	128.07 (13)	H4B—C4—H4C	109.5
N1—C1—N2	123.07 (18)	O2—C7—H7A	110.6
N1—C1—N3	121.99 (17)	C8—C7—H7A	110.5
N2—C1—N3	114.90 (17)	O2—C7—H7B	110.6
N4—C2—O1	127.38 (16)	C8—C7—H7B	110.5
N4—C2—N3	123.38 (16)	H7A—C7—H7B	108.7
O1—C2—N3	109.23 (15)	C7—C8—H8A	109.6
O1—C3—C4	106.13 (16)	C7—C8—H8B	109.4
N5—C5—N6	123.37 (18)	H8A—C8—H8B	109.5
N5—C5—N7	121.73 (17)	C7—C8—H8C	109.4
N6—C5—N7	114.87 (17)	H8A—C8—H8C	109.5
N8—C6—O2	127.38 (17)	H8B—C8—H8C	109.5
N8—C6—N7	123.61 (17)	O2C—Cl1—O1C	110.06 (11)
O2—C6—N7	109.00 (15)	O2C—Cl1—O3C	109.30 (11)
O2—C7—C8	105.74 (16)	O1C—Cl1—O3C	109.01 (10)
C1—N1—H1A	115.1	O2C—Cl1—O4C	110.38 (11)
Cu1—N1—H1A	115.0	O1C—Cl1—O4C	109.74 (10)
C1—N2—H2A	120.0	O3C—Cl1—O4C	108.32 (12)
C1—N2—H2B	120.0	O8C—Cl2—O6C	113.3 (2)
H2A—N2—H2B	120.0	O5B—Cl2—O6B	113.7 (3)
C2—N3—H3C	116.5	O5B—Cl2—O8B	110.5 (3)
C1—N3—H3C	116.5	O6B—Cl2—O8B	110.7 (3)
C2—N4—H4D	115.7	O8C—Cl2—O5C	108.38 (17)
Cu1—N4—H4D	115.8	O6C—Cl2—O5C	111.07 (18)
C5—N5—H5A	115.0	O8C—Cl2—O7C	109.7 (2)
Cu1—N5—H5A	115.1	O6C—Cl2—O7C	107.69 (15)
C5—N6—H6A	120.0	O5C—Cl2—O7C	106.54 (16)
C5—N6—H6B	120.0	O5B—Cl2—O7B	108.5 (3)
H6A—N6—H6B	120.0	O6B—Cl2—O7B	107.5 (3)
C5—N7—H7C	116.4	O8B—Cl2—O7B	105.5 (3)

### Hydrogen-bond geometry (Å, °)

**Table:**

*D*—H···*A*	*D*—H	H···*A*	*D*···*A*	*D*—H···*A*
N1—H1A···O1C^i^	0.88	2.14	3.013 (2)	169
N1—H1A···Cl1^i^	0.88	2.99	3.842 (2)	164
N2—H2A···O4C^i^	0.88	2.37	3.150 (3)	147
N2—H2B···O3C	0.88	2.25	2.981 (3)	141
N3—H3C···O4C	0.88	2.31	3.168 (2)	166
N3—H3C···Cl1	0.88	2.94	3.801 (2)	165
N4—H4D···O7C	0.88	2.20	3.031 (3)	156
N4—H4D···O5B	0.88	2.43	3.244 (4)	154
N5—H5A···O5B	0.88	2.17	3.026 (4)	164
N5—H5A···O7C	0.88	2.28	3.081 (3)	152
N5—H5A···Cl2	0.88	2.95	3.814 (2)	168
N6—H6A···O5C	0.88	2.13	2.950 (3)	155
N6—H6A···O6B	0.88	2.46	3.258 (5)	151
N6—H6B···O6C^ii^	0.88	2.11	2.905 (3)	150
N6—H6B···O7B^ii^	0.88	2.39	3.067 (5)	133
N7—H7C···O6C^ii^	0.88	2.05	2.872 (3)	156
N7—H7C···O6B^ii^	0.88	2.27	3.122 (4)	163
N8—H8D···O1C^i^	0.88	2.29	3.149 (2)	164

Symmetry codes: (i) −*x*+1, *y*+1/2, −*z*−1/2; (ii) −*x*, *y*+1/2, −*z*+1/2.
